# Learning From the Slips of Others: Neural Correlates of Trust in Automated Agents

**DOI:** 10.3389/fnhum.2018.00309

**Published:** 2018-08-10

**Authors:** Ewart J. de Visser, Paul J. Beatty, Justin R. Estepp, Spencer Kohn, Abdulaziz Abubshait, John R. Fedota, Craig G. McDonald

**Affiliations:** ^1^Human Factors and Applied Cognition, Department of Psychology, George Mason University, Fairfax, VA, United States; ^2^Warfighter Effectiveness Research Center, Department of Behavioral Sciences and Leadership, United States Air Force Academy, Colorado Springs, CO, United States; ^3^Cognitive and Behavioral Neuroscience, Department of Psychology, George Mason University, Fairfax, VA, United States; ^4^711 Human Performance Wing/RHCPA, Air Force Research Laboratory, Wright-Patterson Air Force Base, Dayton, OH, United States; ^5^Intramural Research Program, National Institute on Drug Abuse, National Institutes of Health, Baltimore, MD, United States

**Keywords:** error-related negativity (ERN), error processing, error positivity (Pe), automation, event related potentials (ERP), human error, neuroergonomics, anterior cingulate cortex (ACC)

## Abstract

With the rise of increasingly complex artificial intelligence (AI), there is a need to design new methods to monitor AI in a transparent, human-aware manner. Decades of research have demonstrated that people, who are not aware of the exact performance levels of automated algorithms, often experience a mismatch in expectations. Consequently, they will often provide either too little or too much trust in an algorithm. Detecting such a mismatch in expectations, or *trust calibration*, remains a fundamental challenge in research investigating the use of automation. Due to the context-dependent nature of trust, universal measures of trust have not been established. Trust is a difficult construct to investigate because even the act of reflecting on how much a person trusts a certain agent can change the perception of that agent. We hypothesized that electroencephalograms (EEGs) would be able to provide such a universal index of trust without the need of self-report. In this work, EEGs were recorded for 21 participants (mean age = 22.1; 13 females) while they observed a series of algorithms perform a modified version of a flanker task. Each algorithm’s degree of credibility and reliability were manipulated. We hypothesized that neural markers of action monitoring, such as the observational error-related negativity (oERN) and observational error positivity (oPe), are potential candidates for monitoring computer algorithm performance. Our findings demonstrate that (1) it is possible to reliably elicit both the oERN and oPe while participants monitored these computer algorithms, (2) the oPe, as opposed to the oERN, significantly distinguished between high and low reliability algorithms, and (3) the oPe significantly correlated with subjective measures of trust. This work provides the first evidence for the utility of neural correlates of error monitoring for examining trust in computer algorithms.

## Introduction

“Learn from the mistakes of others. You can’t live long enough to make them all yourself."

—Eleanor Roosevelt

With the proliferation of machine learning, complex computer algorithms, and artificial intelligence (AI), there has also been an increase in adverse and unexpected consequences in the use of this technology. For instance, bias for algorithms that have been based on limited training data has already produced several high-profile incidents at companies such as Facebook and Google (Barr, 2015; Economist, 2018). To prevent these kinds of errors and to ensure mutual trust between man and machine or algorithm, there is a great need to provide transparency and understanding with regard to how individuals perceive, respond, and interact with these new forms of technology (Lyons and Havig, 2014; Mercado et al., 2016; Chen et al., 2017).

Historically, expectations between machines and humans have not always been aligned, leading to automation surprises and major accidents (Parasuraman and Riley, 1997). Humans often have *mis-calibrated* trust in automation. That is, they are either too trusting, which leads to over-reliance and complacency, or do not trust enough, which leads to skepticism and disuse of automation (Lee and See, 2004). *Calibrated* trust is when *perceived* trust of automation matches the *actual* trustworthiness of automation (Lee and See, 2004). A number of theories that describe trust in automation have emphasized the relationship between attentional mechanisms and trust. These theories propose that the core mechanism of trust is an interaction between two variables: an initial attentional bias that is updated with observed data about the system (Yeh and Wickens, 2001; Dzindolet et al., 2002, 2003; Madhavan and Wiegmann, 2007b; Rice, 2009; Parasuraman and Manzey, 2010; Hoff and Bashir, 2015). Previous research has established that people usually ascribe a high degree of authority to automation and, as a consequence, use automation advice as a heuristic without necessarily verifying its validity. This phenomenon is known as automation bias (Mosier et al., 1998; Parasuraman and Manzey, 2010). Others have proposed that the general schema for automation is that the agent always performs perfectly (Dzindolet et al., 2003; Madhavan and Wiegmann, 2005, 2007b) and behaves invariantly (Dijkstra et al., 1998). Furthermore, “expert” automated agents are considered more trustworthy than “novice” automated agents (Madhavan et al., 2006). This automation bias therefore affects how systems are monitored and has major consequences when systems fail or do not function optimally (Parasuraman and Riley, 1997).

The measurement of trust varies widely across disciplines primarily because of the wide variety of trust definitions (Erchov, 2017). Given the lack of common measures of trust, it may be fruitful to develop neural measures of trust that are consistent with a neuroergonomic approach (Parasuraman, 2003, 2011; Gramann et al., 2017). Previous research has examined neural correlates of trust between people (Adolphs, 2002; Delgado et al., 2005; King-Casas et al., 2005; Krueger et al., 2007) and while it is expected that the overall trust process for people and automation is similar, it is likely that important and specific differences will emerge between people and machines (Madhavan and Wiegmann, 2007b; de Visser et al., 2016). While contributions toward understanding trust in automated systems from a neuroscientific viewpoint are still limited, recent reviews have pointed to the potential of applying known neural correlates of performance monitoring to the monitoring of machines (Fedota and Parasuraman, 2010; Drnec et al., 2016; Berberian et al., 2017; Somon et al., 2017). Consistent with this idea, a recent study found that false alarm-prone advice activated different brain regions for a human compared to a machine, including the precuneus, posterior cingulate cortex, and temporoparietal junction (Goodyear et al., 2016). Alternatively, miss-prone advice activated salience and mentalizing brain networks differentially for a human compared to a machine (Goodyear et al., 2017). Another study showed that observing errors for humans and machines results in very similar activation in the medial prefrontal cortex (Desmet et al., 2014), although other work showed that this effect can be moderated by human-likeness of the machine agent (Krach et al., 2008). While these studies provide initial evidence of the neural differences between humans and machines, to our knowledge, no study exists that compares neural correlates of trust between humans and machines using electroencephalogram (EEG). Trust is a difficult construct to investigate because even the act of reflecting on how much a person trusts a certain agent can change the perception of that agent. In order to eliminate this metacognitive step, we propose that EEG could provide an index of trust without the need of self-report. Such a measure will be useful in situations when objective assessment of trust is necessary or when it is difficult or undesired to complete a self-report on trust.

A potential candidate for a neural correlate of trust *mis-calibration* is the error-related negativity (ERN), a well-studied event-related potential (ERP) component that is elicited when an individual commits an error (Falkenstein et al., 1991; Gehring et al., 1993). The ERN is a negative-going potential generated in or near the anterior cingulate cortex (ACC) that peaks within 100 ms following an error (Ullsperger et al., 2014b). There is considerable evidence that the ERN indexes a mismatch between predicted and actual outcomes (Falkenstein, 2004; Wessel et al., 2012; Wessel, 2017), and that reduced expectancy for action outcomes is associated with increased amplitude of this component (Fischer et al., 2017). A second error-related component immediately follows the ERN, termed the error positivity (Pe). It has been suggested that, together with the ERN, the Pe may form part of a negative–positive complex. Like the ERN, the Pe is maximal over the frontocentral scalp and there is evidence that it shares a common neural generator with the ERN (Ullsperger et al., 2014b). Whereas the ERN is thought to reflect an automatic, unconscious error detection process, the Pe is believed to be associated with the orienting of attention to the error. Thus, the Pe likely serves as a neural index of error awareness (Ullsperger et al., 2014b; Wessel, 2017).

Quite recently, it has been shown that ERP components comparable to the ERN and Pe can be elicited during the *observation* of an error that was committed by another person or entity (van Schie et al., 2004; Carp et al., 2009). These components, termed the observational error-related negativity (oERN) and observational error positivity (oPe), have similar scalp topographies and neural sources as their performance-related analogs (Koban and Pourtois, 2014). The finding that well-established neural indices of error processing are elicited when observing the actions of others suggests that these indicators may be useful when adopting a neuroergonomic approach to investigate how humans evaluate the performance of automation (Fedota and Parasuraman, 2010).

The present study adapted an established research paradigm (van Schie et al., 2004) to (1) assess whether neural correlates of error monitoring, specifically the oERN and oPe, can be elicited while monitoring the errors of automated agents and (2) evaluate whether these neural markers correlate with the level of trust in those agents. Given that our experimental design is similar to that employed by van Schie et al. (2004) – the only exception being the type and credibility of the agent being observed by participants – the same theoretical explanation for the oERN and oPe can be expected to apply to our investigation. Our general hypothesis was that oERN and oPe signals can index the magnitude of an individual’s trust in the automated algorithm as a function of algorithm credibility and reliability.

In this paradigm, a participant monitors the performance of an automated agent. Historically, the variables credibility (expected performance) and reliability (actual performance) have been critical determinants of human performance with automation (Madhavan et al., 2006; Madhavan and Wiegmann, 2007a,b). Credibility, in this context, is the belief about how well the automated agent is *expected* to perform. In our experiment, reliability presented the accuracy of the automated agent on the Flanker task and thus how well the agent was *actually* performing. Accordingly, we manipulated both credibility and reliability in the same experiment. Given that the oERN is an indication of unconscious error detection and the oPe is likely an indication of error awareness, we expected similar results for both ERP components in our experimental paradigm. Our specific hypothesis was that we predicted an interaction between credibility and reliability such that the largest oERN and oPe would be observed in the highly reliable expert condition and the smallest oERN and oPe would be observed for the unreliable novice condition, with the other two conditions eliciting components of intermediate amplitude. This hypothesis is predicated on the repeated and reliable finding that reduced expectancy for erroneous action outcomes is associated with larger error monitoring signals (Wessel et al., 2012; Fischer et al., 2017). In addition, we hypothesized that oERN and oPe amplitudes would directly correlate with trust in automated algorithms.

## Materials and Methods

### Participants

Twenty-one participants between 18 and 35 years of age (mean = 22.1; 13 females) participated in this study in exchange for either monetary compensation or course credit at George Mason University. Two participants were removed from all analyses due to an insufficient number of trials following EEG rejection. Therefore, 19 participants (mean age = 22.26; 12 females) were incorporated into the analysis. All participants were right-handed, had normal or corrected to normal vision, had no known neurological deficits, and were not taking any medications that affect the nervous system. All participants provided written informed consent after having been explained the experimental procedures. All procedures were approved by the George Mason University Office of Research Integrity and Assurance.

### Experimental Design

Our study was designed with trial accuracy (correct, error), credibility (novice, expert), and reliability (60%, 90%) as within-subject variables to create eight separate conditions. Participants either performed the Flanker task themselves or observed an algorithm perform the task. Credibility was manipulated by having participants read two different stories based on a previous set of experiments (Madhavan et al., 2006; Madhavan and Wiegmann, 2007a). The “expert” story (Flanker–Genius algorithms) described an algorithm that was crafted by the top programmers in the world (see **Appendix A**). The “novice” story (shape-discriminate algorithms) described an algorithm that was crafted by low grade programmers (see **Appendix A**). Reliability was manipulated by varying the response accuracy. In the 60% condition, on average, 6 responses out of 10 were correct. In the 90% condition, on average, 9 responses out of 10 were correct.

### Paradigm and Procedure

Prior to the experiment, participants were informed that they would be evaluating the performance of four computer algorithms that would complete a modified version of the Eriksen flanker task (Eriksen and Eriksen, 1974). The participants were provided with a cover story (see **Appendix A**) that would lead them to believe that two of the algorithms were classified as “experts” at the task (Flanker–Genius algorithms) while the other two algorithms were classified as “novices” (shape-discriminate algorithms). Throughout the experiment, participants would learn that one of the algorithms in each group did not perform as expected. Regardless of how the algorithms were labeled (expert or novice), each group consisted of a good performer (90% credibility algorithm) and a bad performer (60% credibility algorithm). Therefore, the algorithms are described as “Expert 90%,” “Expert 60%,” “Novice 90%,” and “Novice 60%.”

**FIGURE 1:**
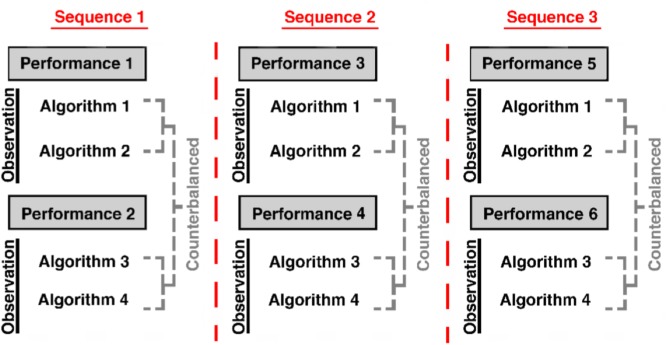
Experiment paradigm describes the order with regard to performance and observation blocks within the experiment. The order of algorithm presentation *within* a sequence was counterbalanced. The algorithms were not repeated until all four algorithms within that sequence had been presented.

The experiment consisted of 18 blocks (six performance blocks; 12 observation blocks) which alternated such that participants performed one block of the task themselves, followed by two blocks in which participants observed an algorithm perform the same task. Each algorithm was observed three times throughout the experiment. However, each of the algorithms was presented once before algorithm presentation was repeated. The order within a single sequence of the four algorithms was counterbalanced across participants (**Figure 1**). Prior to beginning the task, participants briefly practiced both the performance and observation paradigms and were provided with feedback on whether they responded correctly. During the main experiment, they did not receive feedback.

**FIGURE 2:**
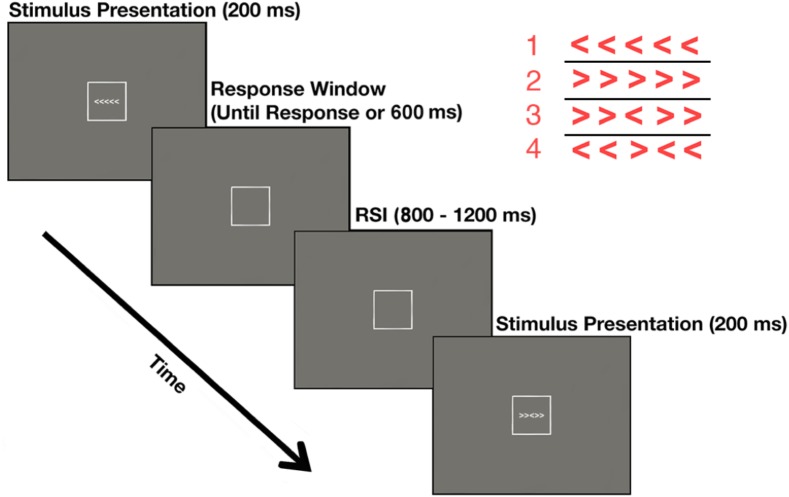
Performance blocks describe the experimental paradigm for blocks in which the participant performed the task themselves. Stimuli are enlarged in the upper right quadrant of the figure for illustrative purposes.

Performance blocks consisted of a modified version of the Erickson flanker task (Eriksen and Eriksen, 1974) in which participants indicated the direction the central target arrow was pointing. The probability of a left or right target arrow as well as the probability of congruent and incongruent stimuli was equiprobable. Participants were instructed to press the “2” key (with their left index finger) if the target was pointing the left or press the “8” key (with their right index finger) if the target was pointing to the right (**Figure 2**). Each block consisted of 160 trials, which were post-experimentally sorted into four bins: Correct Congruent, Correct Incongruent, Error Congruent, and Error Incongruent. During each trial, the stimulus was presented for 200 ms. Participants were required to respond prior to a 600 ms response deadline. Any response that was faster than 150 ms or slower than 600 ms was excluded from all analyses. The response-stimulus interval (RSI) was jittered to occur for 800 to 1200 ms post response. Participants were instructed to weigh the speed and accuracy of their response equally. After each performance block, participants rated their ability to properly identify the direction of the target arrow using a 0 (not at all) to 9 (completely) Likert scale.

Observation blocks consisted of an automated version of the task, in which participants observed and rated each of the four algorithm’s performance on the same flanker task. However, as with previous work on the oERN (van Schie et al., 2004), participants were presented with only the central target arrow of the stimulus array. Participants were told that, behind the scene, the algorithm was still evaluating both the middle arrow and the arrows to either side of it. The algorithm’s “choice” was indicated by the illumination of one of two small circles located below and to either side of the middle arrow. For example, if the arrow pointed to the left and the circle to the left of the arrow illuminated, this indicated that the algorithm made a correct choice. The presentation of a left or right target arrow as well the left and right illuminated circle was equiprobable.

**FIGURE 3:**
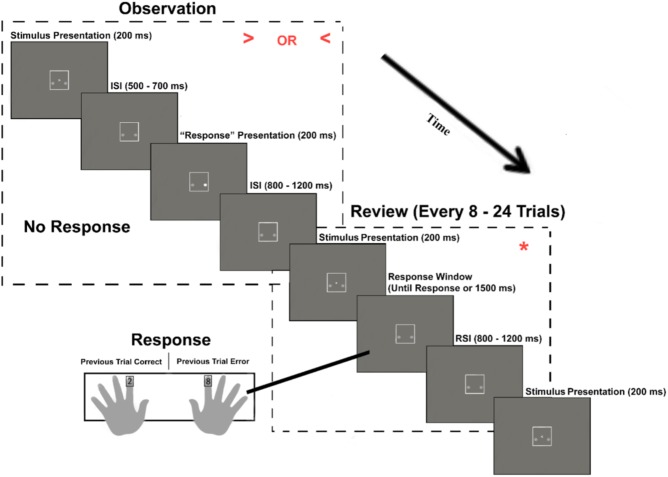
Observation blocks. The experimental paradigm for blocks in which the participant observed an algorithm that performs the task. Stimuli are enlarged in the upper right quadrant of each dashed rectangle for illustrative purposes.

In order to ensure that the participants’ attention to the task was maintained throughout the observation period, participants were prompted every 8–24 trials to complete a “review” trial. During this review, participants were presented with an asterisk, which required them to indicate whether the algorithm’s choice on the preceding trial was correct or incorrect. Participants were instructed to press the “2” key if the algorithm was correct or press the “8” key if the algorithm was incorrect. These response mappings were counterbalanced across participants (**Figure 3**). Observation blocks consisted of 160 trials (with the addition of eight “review” trials). During each trial, the stimulus was presented for 200 ms. An arbitrary response time for the algorithm was set to occur between 500 and 700 ms post stimulus onset. On review trials, any response that was faster than 150 ms or slower than 2000 ms was excluded from all analyses. The RSI was jittered to occur for 800–1200 ms post “response.” After each observation block, participants rated their trust in the observed algorithm’s ability to properly identify the direction of the target arrow using a 0 (not at all) to 9 (completely) Likert scale.

### EEG Data Acquisition and Processing

Electroencephalogram data were recorded through the use of a Neuroscan SynAmps2 amplifier and SCAN 4.3 acquisition software package (Compumedics, Charlotte, NC, United States). More specifically, the data were collected from a 64-channel Ag/AgCl electrode cap, which was arranged according to the extended international 10-20 system. The in-cap reference was located between electrodes Cz and CPz; EEG data were re-referenced offline to the average of M1 and M2 (left and right mastoids). Four electrodes were positioned above and below the left eye and on the outer canthus of each eye in order to monitor electrooculogram (EOG) activity. All EEG data were sampled at 1000 Hz with a bandpass filter of 0.1–70 Hz. The impedance was kept below 5 kΩ for the duration of the experiment.

Processing of EEG data was conducted using the EEGLAB (Delorme and Makeig, 2004) toolbox for the MATLAB programming environment (MathWorks, Natick, MA, United States). Data were detrended to remove large drifts, low-pass filtered at 30 Hz using a Butterworth filter from the ERPLAB plugin (Lopez-Calderon and Luck, 2010), and down-sampled to 500 Hz. On a copy of the original dataset, the data were separated into a series of consecutive 1000 ms epochs and run through an automated rejection of noisy EEG data using a voltage threshold rejection of ±100 aaaV, as well as a spectral threshold rejection using a 50 dB threshold within the 20–40 Hz band using the pop_rejspec function (to remove EMG-like activity; Delorme and Makeig, 2004). If the threshold rejection led to more than 20% of epochs being rejected for a given channel, that channel was removed from all copies of the dataset. The data were then run through independent component analysis (ICA) decomposition (Winkler et al., 2015), in which the identified ICA component weights in the 1 Hz high-pass filtered dataset was copied to the 0.1 Hz high-pass dataset. All further analyses were performed on the 0.1 Hz high-pass dataset.

After the independent components that corresponded to blinks and saccades were rejected, both the performance and observational data were epoched from -200 to 800 ms relative to all stimulus and response markers and run through a more strict automated rejection of noisy EEG data using a voltage threshold rejection of ±75 aaaV and spectral threshold rejection using a 50 dB threshold within the 20–40 Hz band using the pop_rejspec function. Similar to earlier in the processing stream, if the threshold rejection lead to more than 20% of epochs being rejected for a given channel, that channel was removed from all copies of the dataset. Any missing channels were then interpolated using spherical interpolation and all epochs were baseline corrected from -200 to 0 ms.

The average number of trials incorporated into the performance grand-average waveforms was as follows: “Correct-Congruent” (*M* = 412.89; *SD* = 45.74), “Correct-Incongruent” (*M* = 302.53; *SD* = 70.59), “Error-Congruent” (*M* = 10.16; *SD* = 10.29), and “Error-Incongruent” (*M* = 88.21; *SD* = 52.75). However given the scarce number of “Error-Congruent” trials, the performance data were analyzed by collapsing across congruency with the average number of trials in each condition as follows: “Correct” (*M* = 715.42; *SD* = 108.40) and “Error” (*M* = 98.37; *SD* = 59.82). Statistical analysis of the performance ERN, as well as the performance Pe, was conducted using trial accuracy (correct, incorrect) paired-sample *t*-tests. Both components were time-locked to participants’ response during the flanker task and were analyzed at electrode FCZ using a predefined time window of 40 ms for the ERN (4–44 ms) and 60 ms for the Pe (150–210 ms), which were centered on the respective peaks of the grand-average difference (error minus correct) waveform.

The average number of trials incorporated into the observation grand-average waveforms was as follows: “Correct-Expert 90” (*M* = 387.53; *SD* = 40.89), “Correct-Expert 60” (*M* = 255.53; *SD* = 28.12), “Correct-Novice 90” (*M* = 390.74; *SD* = 35.13), “Correct-Novice 60” (*M* = 259.16; *SD* = 23.1), “Error-Expert 90” (*M* = 43.37; *SD* = 5.04), “Error-Expert 60” (*M* = 173.42; *SD* = 17.12), “Error-Novice 90” (*M* = 43; *SD* = 4.45), and “Error-Novice 60” (*M* = 173; *SD* = 15.83). Statistical analysis of the oERN, as well as the oPe, was conducted using 2 × 2 × 2 (trial accuracy by algorithm credibility by algorithm reliability) repeated measures ANOVAs. The oERN and oPe, which were time-locked to the onset of the algorithms’ response during the automated task (illuminated circles), were analyzed at electrode FCZ using a predefined time window of 40 ms for the oERN (202–242 ms) and 60 ms for the oPe (286–346 ms), which were centered on the respective peaks of the grand-average difference (error-correct) waveform.

### Subjective Trust Measurement

An established self-report scale was adapted to measure trust during the task (Lee and Moray, 1992). The single response item was phrased as the following question: “To what extent do you trust the algorithm’s ability to correctly identify the target?” Participants had to respond using a 0 (not at all) to 9 (completely) scale.

### Statistical Analyses

Statistical analyses of the performance ERN, as well as the performance Pe, were conducted using trial accuracy (correct, incorrect) paired-sample *t*-tests. Statistical analyses of the oERN, as well as the oPe, were conducted using 2 × 2 × 2 (trial accuracy by algorithm credibility by algorithm reliability) repeated measures ANOVAs. A 4 × 5 (algorithm by time-point) repeated measures ANOVA was conducted on the subjective trust ratings for the *observation* blocks only. To test if oERN and oPe amplitudes were related to trust rating of the automated algorithms, and whether the relationship can be modulated by the sequence in which the Flanker task was observed, we used the lme4 package in R, which allowed for mixed-effects modeling (Bates et al., 2015). Two linear mixed-effects models predicted oERN and oPe amplitudes separately and contained *trust ratings* as a continuous variable, *sequence* as a dummy coded variable (1, 2, or 3), and their interaction as a moderation effect.

## Results

### Behavior

An average of 296 trials per participant (8.97% of all trials) were removed from the analysis because the response was not made during the allotted response window (i.e., the response latency was less than 150 ms or greater than 600 ms) or more than one response was selected per presentation of a stimulus. Participants were explicitly instructed not to correct for reflexive motor mistakes, but any trials in which residual corrections still took place were removed from the analysis. During performance blocks, the average participant accuracy was 87.08% (congruent trials: *M* = 97.55%, *SD* = 3.19%; incongruent trials: *M* = 76.62%, *SD* = 14.94%). A paired-sample *t*-test revealed a significant effect of trial accuracy [*t*(18) = 11.35, *p* < 0.001, *d* = 1.82] in which incorrect responses (*M* = 393.44; *SE* = 5.51) were faster than correct responses (*M* = 440.75; *SE* = 6.27). In addition, when evaluating only correct responses, a paired-sample *t*-test revealed an effect of congruency [*t*(18) = -15.32, *p* < 0.001, *d* = -2.29], in which responses were faster for congruent (*M* = 407.46; *SE* = 6.83) than incongruent (*M* = 474.03; *SE* = 6.44) trials. These findings replicate the typical behavioral responses observed for the Eriksen flanker task (Eriksen and Eriksen, 1974).

During observation blocks, accuracy of the algorithm was set at either 90 or 60% (depending on which algorithm was executing the task) and the “response time” was set to vary between 500 and 700 ms (*M* = 609.57; *SD* = 6.37).

### ERP Components

**FIGURE 4:**
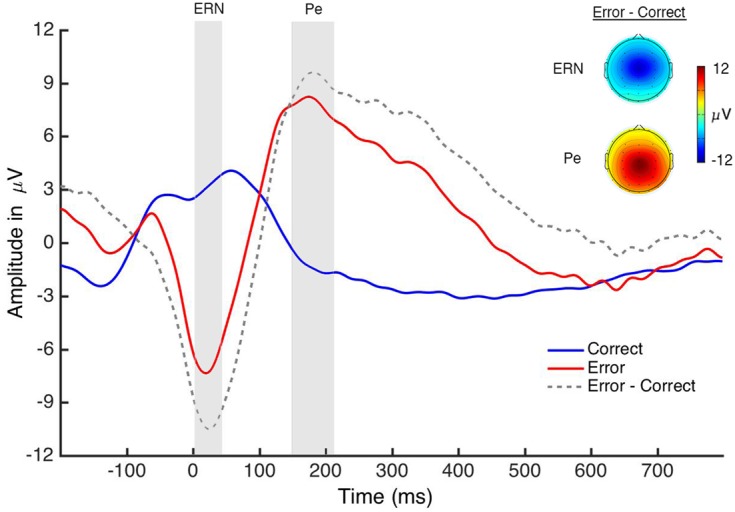
Performance ERPs. The graph displays the response-locked ERN and Pe that were generated when *participants* performed the task. The gray boxes indicate the analysis window for each component.

For the performance ERN (**Figure 4**), a paired-sample *t*-test revealed an effect of accuracy [*t*(18) = 7.03, *p* < 0.001, *d* = 2.08] in which amplitude was larger (more negative) on error trials (*M* = -6.82; *SE* = 0.91) compared to correct trials (*M* = 3.23; *SE* = 1.27; see **Table 1**). For the performance Pe (**Figure 4**), a paired-sample *t*-test revealed an effect of accuracy [*t*(18) = -4.57, *p* < 0.001, *d* = -1.45] in which amplitude was larger (more positive) on error trials (*M* = 7.84; *SE* = 1.77) than on correct trials (*M* = -1.31; *SE* = 1.01; see **Table 1**). This pattern of results is consistent with a large extant literature on these components (Steinhauser and Yeung, 2010). Additionally, the frontocentral scalp topographies of these components are consistent with prior reports (Ullsperger et al., 2014b).

**Table 1:** Performance and observational event-related potentials.

**Performance ERP components**							
**ERN**				**Pe**			
**Variable**	**Mean (*SE*)**	***t*(18)**	***p***	**Variable**	**Mean (SE)**	***t*(18)**	***P***
Accuracy		7.03	<0.001	Accuracy		-4.57	<0.001
Correct	3.23 (0.27)			Correct	-1.31 (1.01)		
Incorrect	-6.82 (0.91)			Incorrect	7.84 (1.77)		
**Observational ERP components**							
**oERN**				**oPe**			
**Variable**	**Mean (*SE*)**	***F*(1,18)**	***p***	**Variable**	**Mean (*SE*)**	***F*(l,18)**	***P***
Accuracy		8.74	0.008	Accuracy (Acc)		18.46	<0.001
Correct	2.03 (0.45)			Correct	0.71 (0.35)		
Incorrect	-0.01 (0.78)			Incorrect	4.95 (0.98)		
				Reliability (Rel)		6.01	0.025
				90%	3.24 (0.65)		
				60%	2.42 (0.47)		
				Acc ^∗^ Rel		22.13	<0.001
				Correct			
				90%	0.2 (0.39)		
				60%	1.23 (0.34)		
				Incorrect			
				90%	6.29 (1.23)		
				60%	3.61 (0.81)		

*Tabulated values for statistics performed on event-related potentials during their respective analysis windows. Values for means and standard errors are in aaaV amplitudes.*

**FIGURE 5:**
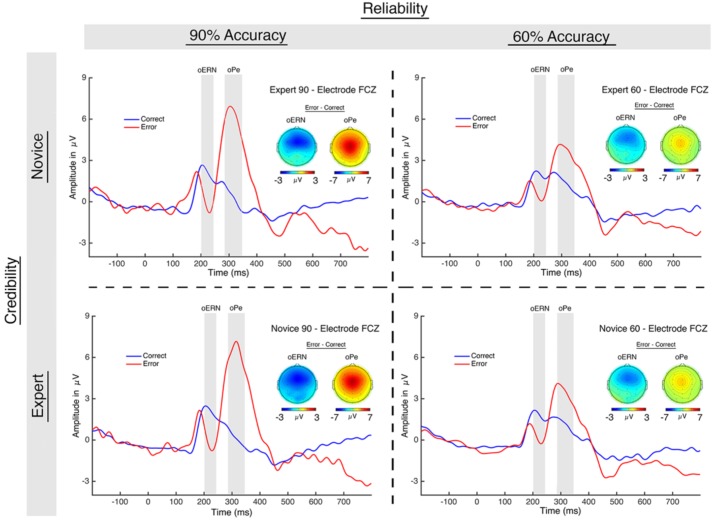
Observation ERPs. The graph displays the oERN and oPe, which were time-locked to each algorithm’s “response.” The participants did not perform the task themselves – they just evaluated the reliability of the algorithm. The gray boxes indicate the analysis window for each component.

For the oERN (**Figure 5**), a 2 (trial accuracy: correct, error) × 2 (credibility: novice, expert) × 2 (reliability: 60%, 90%) repeated-measures ANOVA revealed a main effect of accuracy [*F*(1,18) = 8.74, *p* = 0.008, η_P_^2^ = 0.327], in which amplitude was larger (more negative) on error trials (*M* = -0.01; *SE* = 0.78) than on correct trials (*M* = 2.03; *SE* = 0.45; see **Table 1**). All other effects failed to reach significance (*p* > 0.18).

For the oPe, a 2 (trial accuracy: correct, error) × 2 (credibility: novice, expert) × 2 (reliability: 60, 90%) repeated-measures ANOVA revealed a main effect of accuracy [*F*(1,18) = 18.46, *p* < 0.001, η_P_^2^ = 0.506], in which amplitude on error trials (*M* = 4.95; *SE* = 0.98) was larger (more positive) than on correct trials (*M* = 0.71; *SE* = 0.35; **Figure 5**). There was also a main effect of reliability [*F*(1,18) = 6.01, *p* = 0.025, η_P_^2^ = 0.250], in which the oPe was larger (more positive) for the 90% performing algorithms (*M* = 3.24; *SE* = 0.65) than the 60% performing algorithms (*M* = 2.42; *SE* = 0.47). Lastly, there was a significant accuracy by reliability interaction [*F*(1,18) = 22.13, *p* < 0.001, η_P_^2^ = 0.551] in which oPe amplitude on error trials was larger (more positive) than on correct trials for both 90% (*p* < 0.001, *d* = -1.52) and 60% (*p* = 0.008, *d* = -0.84) performing algorithms (see **Table 1**). All other effects failed to reach significance (*p* > 0.44).

The scalp topographies for the oERN and oPe are very similar to those of the ERN and Pe, although the Pe has a more central distribution than the oPe. We also note that the waveforms of both the performance and observation ERPs are strikingly similar. Although it is not possible to infer neural sources based on scalp topographies, the similar spatial distribution and time course of the performance and observation ERPs suggests that they likely reflect comparable neural processes.

### Analysis of Algorithm Trust Ratings

Between-block ratings with regard to the participant’s ability to perform the task themselves (examined post-performance blocks) as well as between block ratings with regard to the participant’s trust in the algorithm to perform the task (examined post-observation blocks) were recorded. Although the ratings with regard to the *performance* blocks are not discussed here, a 4 × 5 (algorithm by time-point) repeated measures ANOVA on the *observation* ratings revealed a main effect of algorithm [*F*(1,18) = 34.024, *p* < 0.001, η_P_^2^ = 0.654], as well as an algorithm by time-point interaction [*F*(1,18) = 8.944, *p* < 0.001, η_P_^2^ = 0.332].

**FIGURE 6:**
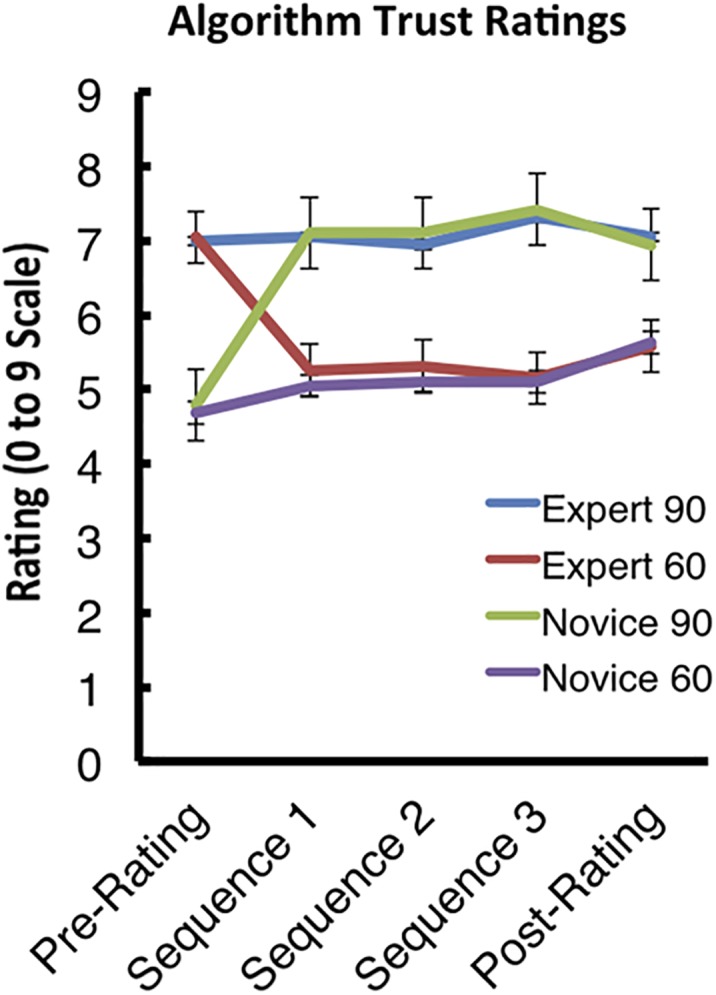
Algorithm trust ratings. The graph displays the pattern of trust ratings over the course of the experiment. Error bars indicate the standard error of the mean (SEM).

However, the main effect of time-point failed to reach significance (*p* = 0.731). Interestingly, the only difference in pattern for rating the algorithms over time occurred after the participants’ first interaction with each algorithm (**Figure 6**). This indicates that, although the cover-stories were successful in establishing the expert and novice algorithm credibility initially, the participants very quickly reached the end-state with regard to determining the true objective performance of each algorithm.

### Mixed-Linear Effects Analyses of ERP Component Magnitude and Trust Ratings

To investigate the relationships between ERP amplitudes and subjective ratings, we used a linear mixed model to test if subjective trust ratings were related to oERN and oPe amplitudes and if the ERP-trust ratings relationships were modulated as a function of algorithm observation sequence.

**FIGURE 7:**
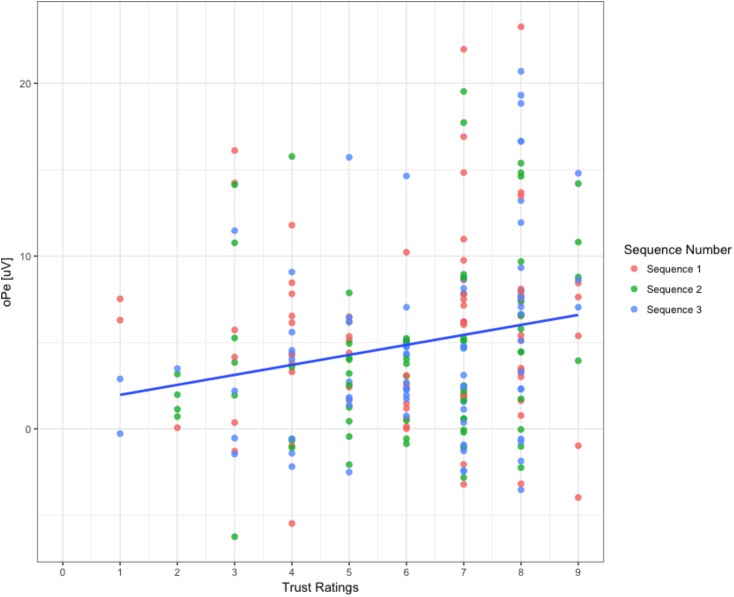
Correlation between oPe magnitude and trust ratings. The graph portrays all algorithm types for the three sequences when participants observed the algorithms. The line of best fit is displayed across all three sequences.

Results of the first model that predicted oPe amplitudes accounted for 65% of the variance (*R*^2^ = 0.65) and revealed that *trust ratings* was a significant predictor of oPe [*b* = 1.01, β = 0.18, *SE* = 0.41, *t*(211.5) = 2.41, *p* = 0.01], which suggests that oPe amplitudes increased as *trust ratings* increased (see **Figure 7**). The dummy coded variable of *sequence* showed a significant mean difference between the *sequence* 1 and *sequence* 2 [*M*_Sequence1_ = 5.56 uV, *M*_Sequence2_ = 4.37 uV, *SE* = 0.54, *t*(204) = -2.17, *p* = 0.03], which suggests that the mean amplitudes were lower overall for sequence 2 compared to sequence 1. However, no significant mean differences between *sequence* 1 and 3 were evident [*M*_Sequence1_ = 5.56 uV, *M*_Sequence3_ = 4.92 uV, SE = 0.54, *t*(204) = -1.15, *p* = 0.24].

The interaction term between *trust ratings* and *sequence* 2 was not significant [*b* = 0.71, β = 0.13, *SE* = 0.54, *t*(204.5) = 1.31, *p* = 0.19], showing that the *oPe-trust rating* relationship did not differ significantly between *sequence* 1 and 2. Similarly, the interaction term between *trust ratings* and *sequence* 3 showed a non-significant difference [*b* = 0.79, β = 0.14, *SE* = 0.54, *t*(204.6) = 1.45, *p* = 0.14] suggesting that the oPe-trust rating relationship in sequence 1 compared to sequence 3 was not different.

Analysis of the oERN model accounted for 55% of the variance (R^2^ = 0.55) and revealed that *trust ratings* did not predict oERN amplitudes [*b* = -0.16, β = -0.03, *SE* = 0.36, *t*(213.0) = -0.45, *p* = 0.65]. The dummy coded variable showed no mean differences between *sequence* 1 and *sequence* 2 [*M*_Sequence1_ = 0.01 uV, *M*_Sequence2_ = -0.21 uV, *SE* = 0.47, *t*(204.1) = -0.47, *p* = 0.63] or between *sequence* 1 and 3 [*M*_Sequence1_ = 0.01 uV, *M*_Sequence3_ = 0.3 uV, *SE* = 0.47, *t*(204.1) = 0.62, *p* = 0.54]. The interaction terms also showed no modulation of the oERN-trust relationship between *sequence* 1 and *sequence* 2 [*b* = -0.09, β = -0.02, *SE* = 0.47, *t*(204.8) = -0.2, *p* = 0.83], or *sequence* 1 and *sequence* 3 [*b* = -0.05, β = -0.01, *SE* = 0.47, *t*(204.9) = 0.12, *p* = 0.9].

## Discussion

The primary goal of the present study was to determine whether neural indices of error monitoring could be observed while participants monitored the performance of a computer algorithm. We demonstrate for the first time that the oERN and oPe were reliably elicited when computer algorithms committed errors while performing the Eriksen flanker task. This finding elaborates on the work of van Schie et al. (2004), who demonstrated the oERN using human agents. In addition, we hypothesized that the oERN and oPe would be sensitive to differences in credibility and reliability of the algorithms. We found that the oPe was modulated by the reliability, but not the credibility, of the algorithms. In contrast, we did not find a similar effect for the oERN. Questionnaires taken during the experiment further confirmed that participants quickly converged on the reliability levels of each of the algorithms and quickly ignored the credibility levels. Finally, the mixed-linear effects results revealed that oPe amplitudes significantly and positively correlated with subjective trust ratings across the three sequences.

Our study expands on van Schie et al. (2004) seminal work in several important ways. First, the agents used in the present study were computer algorithms instead of other humans in the same room. This is an important elaboration of the original study because our findings show that neural correlates of error detection extend not just to the observation of other people, but also to computer agents. Second, we have linked, for the first time, a neural mechanism of error monitoring and awareness as a key driver of subjective assessments of trust in computer algorithms. Prior studies have not established this link. Third, we have manipulated algorithm reliability rates and shown that these rates directly affect oPe amplitudes. Lastly, we have established that credibility as manipulated by a background story had a negligible effect on performance compared to the reliability of an algorithm.

### What Do oPe and oERN Reflect?

The oPe and oERN, as well as their performance analogs, are generally believed to reflect error salience, which increases as a function of the magnitude of the mismatch between the expected and actual action outcomes. Compelling evidence for this notion has been provided in a recent large-scale study showing that ERN amplitude increases as the frequency of errors is diminished (Fischer et al., 2017). This finding is consistent with work suggesting that the ERN indexes the automatic processing of unexpected events, irrespective of whether those events are unexpected stimuli or erroneous actions (Wessel et al., 2012; Wessel, 2017). Similarly, the Pe has been suggested to be an index of reflexive attentional orienting to errors (Ullsperger et al., 2014a). Given that attentional orienting is stronger to unexpected events, it seems reasonable to suggest that errors, which are typically infrequent (and therefore unexpected), would evoke an oPe of greater amplitude (Ullsperger et al., 2014a). Thus, given that the reliable algorithms made errors at a lower frequency than unreliable algorithms, it is not surprising that the oPe was larger for reliable algorithms. Although the oERN would be expected to behave in a similar fashion to the oPe, it is possible that a reduced signal to noise ratio, which would be expected for this smaller component (at least in the observation condition), precluded detecting a significant effect.

### Neuroergonomics of Trust: Initial Evidence for Neural Correlates of Trust Calibration

The important discipline of neuroergonomics attempts to connect neural mechanisms with broader cognitive constructs in human factors and ergonomics fields (Parasuraman, 2003). Consistent with this effort, our findings of neural indices of observational error monitoring and positive correlation of one of these indices (oPe) with subjective trust ratings provide the first evidence for candidate neural mechanisms of established findings in the automation literature as well as automation theories (Fedota and Parasuraman, 2010).

A major finding in the automation literature is that the salience of an automation error is directly related to the perceived trustworthiness of the automation. This phenomenon manifests itself in several ways. For example, operators may be startled when an automated system, such as an automated pilot, does something that does not match their current mental model of the system, an effect known as automation surprise (Sarter et al., 1997). A similar effect occurs when operators notice automation errors during tasks that they can easily perform themselves (Madhavan et al., 2006). The ease of the task makes automation errors stand out, thus increasing their saliency. Finally, there is the *first failure effect*, the notion that the first automation failure experienced with automation has a strong anchoring effect on subsequent interaction with automated system (Wickens and Xu, 2002; Rovira et al., 2007). We believe that each of these phenomena is driven by the mismatch in mental models, unexpected events, or high saliency, and that a neural mechanism behind these phenomena is captured by the oERN/oPe response detected in the present study. In our study, the finding that rare errors produced a significantly larger oPe demonstrates that these effects produce more awareness at the neural level. The mixed-linear effects analysis of the oPe with the subjective trust ratings is direct evidence that error awareness is a critical mechanism that predicts trust in the algorithm.

An additional observation by Madhavan et al. (2006) suggests that automation failures on easy tasks (such as the Eriksen flanker task) are more detrimental to performance than difficult tasks – primarily because errors are more noticeable. Our study supports the idea that the oPe is a neural driver of this monitoring process because our results demonstrated that this component varied with the salience of the errors as manipulated by the reliability of the algorithm. Furthermore, previous research has suggested the high initial expectations of automation performance, known as automation bias, may induce a “fall from grace” with low reliability automated systems. For instance, if an operator has an initially high expectation of automation performance, but observes that automation actually has low performance, the trustworthiness of this automation takes an extra hit, above and beyond what would be explained by just low reliability performance. While our study showed a strong reliability effect, we did not find this interaction between credibility and reliability. A possible reason for not observing an interaction between credibility and reliability may have been due to the fact that our participants quickly converged on the true reliability levels of the algorithms. The Eriksen flanker task leaves little ambiguity and is a clear-cut task. Any attempt to infuse credibility through stories was therefore more complicated. Introducing more difficult tasks with lower signal-to-noise ratio will make it more likely that credibility will play a lasting role in the experiment. Increasing uncertainty of the stimulus or increasing attentional load may also achieve this effect.

Finally, our findings present evidence for the performance or ability dimension of trustworthiness (Mayer et al., 1995; Lee and See, 2004) that reflect the ability of an agent to perform adequately on a task. Our assessment did not include dimensions of integrity or benevolence. However, it is possible that the ACC, the presumed neural generator of the oERN and oPe, is involved in a more generalized capability of predicting the behavior of an agent and reflecting a trust policy toward that agent. Previous research has shown an “intention to trust” signal in the ACC and caudate nucleus (Delgado et al., 2005; King-Casas et al., 2005). A more general theory of trust based in neuroscience will also need to include an explanation of dimensions of trustworthiness such as integrity and benevolence.

### Applications

The current findings suggest that the oPe might provide a more reliable neural index when monitoring artificial agents, although it is possible that the oERN may be useful in different contexts (van Schie et al., 2004). Indeed, the ERN has been used in real-time passive brain–computer interface (BCI) applications or for controlling a robotic arm such as the Baxter robot (Bryk and Raudenbush, 1987; Chavarriaga and del Millán, 2010; Zander et al., 2010, 2016; Zander and Kothe, 2011; Chavarriaga et al., 2014; Grissmann et al., 2017; Salazar-Gomez et al., 2017). In a study similar to our task, participants gazed at a robot that decides between targets (Salazar-Gomez et al., 2017). The robot used its arm to reach for the target either correctly or incorrectly. In this paradigm, the EEG signal was processed and classified based on the features of the error potentials. Using this machine learning approach, individual error potentials could be used to provide feedback to the robot on a single-trial basis in a closed-loop scenario, allowing the robot arm to switch to the correct target in the middle of the reach. By extending this work to cognitive tasks, such as driving and flying, it may also be possible to use these error signals to serve as an early indicator of a mental model mismatch between human and machine such as automation surprise during performance monitoring in a work setting (Sarter et al., 1997), as a real-time measure of trust to drive adaptive automation approaches (Byrne and Parasuraman, 1996; Scerbo, 1996, Moray, 2000; Scerbo et al., 2003; Prinzel et al., 2003; de Visser and Parasuraman, 2011) or during social interactions between robots and people (Abubshait and Wiese, 2017; Mirnig et al., 2017; Wiese et al., 2017). An ERP signal may be particularly useful in time-critical situations, when subjective reports cannot be completed or when subjective reports are not reliable. Collectively, the research to date suggests that neural indices of error monitoring are good candidates for indicating whether either the machine or the human needs assistance with current task performance. While this research has already demonstrated the ability to detect error signals without any other data, the accuracy of the general moment-to-moment user model can be updated with additional sources of data such as a person’s profile, mood, and other tendencies. The neural measurement of error detection and awareness can thus serve as a useful and objective proxy of an algorithm’s perceived trustworthiness.

### Limitations

This study had a number of limitations. First, we examined only two reliability conditions, namely the 60 and 90% conditions. The primary reason we chose these reliability levels was to have algorithms perform distinctly different while both attaining enough errors and keeping algorithm performance above chance. Future studies could examine further whether the oERN/oPe amplitudes vary consistently with the degree of reliability anywhere between 0 and 100%. Evidence that such sensitivity of amplitude exists has been provided by a study that mapped out how close participants were to achieving their goal by passively monitoring error responses to a dot moving on screen in various directions. In this study, participants exhibited a consistently stronger error signal when they were further removed from their goal (Zander et al., 2016). Second, our study had a small sample size. Despite the small sample size, our effect sizes were large which alleviates concerns of low power. We also employed statistical methods that are designed to account for small samples such as mixed linear effects analyses. Third, this study did not allow us to quantify the consequences of the magnitude of oPe on subsequent choice to interact or comply with a given agent in the future. Compliance with agent recommendations is one of the primary behavioral indicators of trust in automation research in more ecologically valid tasks. For instance, a popular task to examine this trust in automation cycle is the bag-screening task. In this task, participants can screen for dangerous objects themselves and follow an automated agent’s recommendation (Madhavan et al., 2006; Madhavan and Wiegmann, 2007a; Merritt and Ilgen, 2008; Merritt et al., 2013, 2014, 2015; Pop et al., 2015). A complete neural explanatory mechanism of trust calibration in automated agents would need to include the ERP profile of the observation of the automation’s performance, the evaluation of the automated agent’s decision recommendation as well as the feedback on the consequence of either complying or not complying with the advice of the agent. It is also important to validate this work in more ecologically valid tasks to determine whether a reliable signal can be extracted with varying task parameters in different work domains. Lastly, a wider range of accuracy measures could be employed to find the dose-response relationship specifying when errors become more salient for a given credibility expectation. For example, if automation performance was ∼72% for incompatible trials, perhaps an agent that performs just over or under that threshold elicits different ERPs.

Despite these limitations, the primary and main contribution of our work is that we have identified a specific neurobiological mechanism for subjective trust evaluations of trust in automated agents. This discovery will help enable the development of objective measurement of trust in automated systems, a significant improvement over subjective evaluations of trust.

## Conclusion

Neural indices of error monitoring provide a novel, theoretically grounded approach for monitoring the behaviors of other agents. We have demonstrated that this approach is valid for monitoring of and calibrating trust in computer algorithms. Our work is consistent with a neuroergonomic approach in which we combine both neuroscience and ergonomic theories to explain the brain at work.

## Ethics Statement

All procedures were approved by a University Institutional Review Board of George Mason University (protocol # 920040-3) and participants provided written informed consent in accordance with the Declaration of Helsinki.

## Author Contributions

EdV, JF, SK, PB, andCMcontributed to the conception and design of the study. PB collected the data. PB and AA analyzed the data. All authors contributed to the interpretation of the findings of the study. EdV, PB, AA, and CM drafted the manuscript.

## Conflict of Interest Statement

The authors declare that the research was conducted in the absence of any commercial or financial relationships that could be construed as a potential conflict of interest.

## Appendix A

The following script was used to manipulate credibility:

Throughout this experiment, you will observe the performance of two classes of computer algorithms as they perform a modified version of the Eriksen Flanker task.

### Flanker-GENIUS Algorithms

You will observe the performance of two expert-class computer algorithms called Flanker-GENIUS (Version Gamma and Version Delta), which are “automated feature discrimination classifiers” that are able to consistently detect the orientation of the Eriksen Flanker arrows. The algorithms are built upon 15 years of research on how humans perform the Eriksen Flanker task.

Flanker-GENIUS was developed through a relationship between two devoted academic labs at the Massachusetts Institute of Technology (MIT). The combined intelligence of a dozen professors and PhD students with expertise in computer vision and machine learning contributed to the final award-winning software product.

The Flanker-GENIUS algorithms have been thoroughly trained on an extensive database of shapes and characters under a wide variety of noise conditions such as fuzziness, incompleteness, and visual distortions. The algorithms continue to improve themselves through thousands of sessions of inferential learning. In their current state, the tools are consistently dependable and literate in a wide variety of features: they can detect individual shapes, arrows, and letters in very complex images.

Flanker-GENIUS algorithms have recently been deployed to test and create increasingly secure CAPTCHAs, images that distort letters and words to determine whether users are humans or spam-generating robots. They will soon be available to the community as a free service.

### Shape-DISCRIMINATE Algorithms

You will observe the performance of two novice-class computer algorithms called shape-DISCRIMINATE (Version Kappa and Version Theta), which are “automated feature discrimination classifiers” that were designed to discriminate between simple shapes, such as triangles and squares. The algorithms are unfinished prototypes that are still experiencing some technical issues.

shape-DISCRIMINATE is a group project by eight undergraduate students in the computing department at a Midwestern technical college. The students have a variety of academic backgrounds, including visual arts. The algorithm is part of a class project on computer programming. Their project adapted a discarded code-base from an online catalog.

Shape-DISCRIMINATE algorithms have had limited training on simplistic clear images with uncomplicated shapes and no visual noise. They cannot process images that contain distortions or complex characters, and remain relatively unsophisticated in their attempt to determine whether a shape is a triangle or a square. The students are attempting to fix software bugs to conduct further training with the tools. In their current state, the tools occasionally malfunction and require human assistance.

The students will soon deploy the shape-DISCRIMINATE algorithms to attempt to find and defeat unsecured online CAPTCHAs, images that distort letters and words to determine whether users are humans or robots. Shape-DISCRIMINATE algorithms will be made available as a paid tool after all bugs are fixed.
